# Polyfunctional natural killer cells with a low activation profile in response to Toll-like receptor 3 activation in HIV-1-exposed seronegative subjects

**DOI:** 10.1038/s41598-017-00637-3

**Published:** 2017-04-03

**Authors:** Josenilson F. Lima, Luanda M. S. Oliveira, Nátalli Z. Pereira, Alberto J. S. Duarte, Maria N. Sato

**Affiliations:** Laboratory of Dermatology and Immunodeficiencies, LIM-56, Department of Dermatology, Institute of Tropical Medicine, University of São Paulo, São Paulo, Brazil

## Abstract

Natural killer (NK) cells are the main mediator of the cytotoxic response in innate immunity and may be involved in resistance to HIV-1 infection in exposed seronegative (ESN) individuals. Toll-like receptor (TLR) signalling is crucial for NK cell activation. Here, we investigated the polyfunctional NK cell response to TLR3 activation in serodiscordant couples. ESN subjects showed increased IFN-γ and CD107a expression in both NK subsets, CD56^bright^ and CD56^dim^ cells, in response to stimulation with a TLR3 agonist, while expression was impaired in the HIV-1-infected partners. TLR3-induced expression of IFN-γ, TNF and CD107a by polyfunctional CD56^bright^ NK cells was more pronounced in ESN individuals than that in healthy controls. Activated NK cells, as determined by CD38 expression, were increased only in the HIV-1-infected partners, with reduced IFN-γ and CD107a expression. Moreover, CD38^+^ NK cells of the HIV-1-infected partners were associated with increased expression of inhibitory molecules, such as NKG2A, PD-1 and Tim-3, while NK cells from ESN subjects showed decreased NKG2A expression. Altogether, these findings indicate that NK cells of ESN individuals were highly responsive to TLR3 activation and had a polyfunctional NK cell phenotype, while the impaired TLR3 response in HIV-1-infected partners was associated with an inhibitory/exhaustion NK cell phenotype.

## Introduction

Some individuals remain HIV-1-seronegative despite repeated unprotected exposure to the virus^1^. These individuals are usually defined as HIV-1-exposed seronegative (ESN) subjects in study cohorts with different exposure profiles: sex workers, children born to HIV-seropositive mothers, intravenous drug users, health care workers accidentally exposed to HIV, and homosexual or heterosexual subjects who have a history of unprotected sex with their seropositive partners^2^. Resistance to HIV-1 infection in ESN individuals has been associated with the presence of antigen-specific immune responses as well as with components of innate immunity^3^. Increased natural killer (NK) cell activity has been correlated with protection from infection in several high-risk cohorts of ESN subjects, a finding that suggests the involvement of the innate immune response in resistance to HIV-1 infection^4^.

NK cells are considered the founding member of group 1 innate lymphoid cells (ILC1), which show immunological characteristics of lymphoid developmental origin, the absence of clonally rearranged antigen receptors, and an activation profile that requires T-bet and leads to rapid cytokine production, including IFN-γ^5, 6^. Two major subtypes of functionally distinct NK cells include CD56^bright^CD16^−^ (CD56^bright^) cells, which represent approximately 10% of circulating NK cells and are predominantly involved in cytokine and chemokine secretion, and CD56^dim^CD16^+^ cells^7^, which represent the remaining 90% of circulating NK cells and are responsible for recognition and lysis of target cells^8, 9^. The L-selectin molecule (CD62L) mediates homing of leukocytes to lymphoid organs, and CD56^dim^CD62L^+^ cells represent a unique subset of mature, polyfunctional NK cells that affect the magnitude of the local NK cell response to murine viral infection^7, 10^. These polyfunctional cells have the ability to produce IFN-γ after cytokine stimulation, proliferate *in vivo* during viral infection, and kill target cells upon engagement of activating receptors.

The NK cells of ESN drug users showed high cytolytic potential and produced IFN-γ, TNF-α, and β-chemokines when in contact with the K562 cell line^11^. In addition, NK cells from the ESN subjects produced high levels of IFN-γ in response to activation with phorbol myristate acetate and ionomycin^12^. The absence of human leukocyte antigen (HLA) binding to inhibitory killer-cell immunoglobulin-like receptors (KIRs), including KIR2DL2, KIR2DL3 and KIR3DL1, leads to a reduced activation threshold of NK cells from ESN individuals and has been associated with resistance to HIV-1 infection^13^.

In contrast, chronic HIV-1 infection alters the population distribution and functional capacity of NK cells. The chronic activation receptor CD38 is an ectoenzyme expressed on CD8^+^ T cells and is associated with progression to AIDS in chronically HIV-1-infected patients, even in those treated with antiretroviral therapy (ART)^14, 15^. Moreover, NK cell activation through CD38 is increased in HIV-1-infected subjects progressing to AIDS, but not in elite and viremic controllers, and is associated with viremia and disease progression markers in both HIV-1 and HIV-2 infections^16, 17^. Whether the NK CD38^+^ cells in HIV-1 infection are associated with altered expression of inhibitory/exhaustion molecules, such as NKG2A, PD-1, and Tim-3, is unclear. Few cytotoxic NK cells and high NKG2A expression have been observed in patients with late-stage HIV infection^18^. The expression of Tim-3, a type I transmembrane protein, has been implicated both in activation and inhibition of immune responses^19, 20^, in the induction of apoptosis of Tim-3–bearing cells through interactions with galectin-9^21^ and suppression of cell-mediated cytotoxicity^22^.

NK cells possess receptors allowing them to sense and respond to viral and bacterial patterns, including Toll-like receptors (TLRs)^23^. Viral nucleic acids, such as viral DNA, dsRNA, and ssRNA, can activate nucleic acid-sensing TLRs, including TLR3, TLR7, TLR8 and TLR9, to prevent viral invasion of cells via induction of type I IFN and production of antiviral factors^24^. TLR stimulation in ESN individuals induced a robust release of immunologic factors that can influence adaptive antiviral immune responses^25^. A TLR3 agonist, polyinosinic-polycytidylic acid [Poly(I:C)], a synthetic mimetic of viral RNA, significantly augmented NK cell-mediated cytotoxicity in healthy individuals^26^. A common TLR3 polymorphism in ESN individuals suggested the potential use of TLR3 triggering in HIV-1 immunotherapy^27^.

In this study, we evaluated the TLR3-induced activation of NK cells and assessed CD62L and CD38 expression in NK cell subsets to verify the polyfunctional response of NK cells in ESN individuals and their HIV-1-infected partners. Moreover, we examined whether CD38^+^CD62L^+^ NK cells were less responsive to TLR3 activation and could be associated with inhibitory/exhaustion markers, such as NKG2A, PD-1 and Tim-3. Elucidation of the underlying mechanisms of naturally manifested resistance against HIV-1 infection in ESN individuals has significant clinical implications for preventive and therapeutic strategies.

## Materials and Methods

### Study population

HIV-1-serodiscordant couples were enrolled from an outpatient clinic at the Emílio Ribas Infectious Diseases Institute in São Paulo, from the Ambulatory Service (ADEE/3002) of the Department of Secondary Immunodeficiency Clinic of the Clinical Hospital, University of São Paulo Medical School (HC/FMUSP) and from the Reference Center and Treatment in STD-AIDS in São Paulo, Brazil. ESN individuals (n = 20), HIV-1-infected partners of ESN individuals (n = 20), and healthy donors not infected with HIV-1 (n = 20) were enrolled in this study. A mean relationship duration of 13 years with a single partner was reported by homosexual (n = 6) and heterosexual (n = 14) couples. Couples reported participating in vaginal, anal and oral sex, including 5 episodes of unprotected sexual intercourse at a frequency of 3–4 times per month^28, 29^. Inclusion criteria consisted of an age over 18 years, reported participation in unprotected sex and having a single partner for over 1 year. The ESN cohort was seronegative at the studied time point. All HIV-1-infected individuals were receiving ART, and detectable viral loads (VLs) were found in 4/20 individuals. Exclusion criteria consisted of the use of immunosuppressant or immune-modifying drugs and pregnancy. This study was approved by the São Paulo University Institutional Use Committee (CAPPesq n° 0683/09), and informed consent was obtained from all subjects. All experimental protocols within this study were performed in accordance with the Ethics Committee of this institution.

### Flow cytometry analysis of peripheral blood

For analysis of the activation/exhaustion/inhibition markers in the NK cells of the peripheral blood, venous blood was collected in EDTA-anticoagulated tubes, and staining was performed using the following antibodies: CD3 (BV605/Clone: SK7), CD19 (Horizon V500/Clone: HIB19), CD16 (APC-Cy7/Clone: 3G8), CD56 (Alexa 700/Clone: B159), CD62L (CF594/Clone: Dreg 56), CD38 (FITC/Clone: HIT2), and PD-1 (PerCP-Cy5.5/Clone: EH12.1) from BD Pharmingen (San Jose, CA, USA) and NKG2A (PE/Clone: 131411; R&D, Minneapolis, MN, USA) and Tim-3 (PE-Cy7/Clone: F38-2E2; Biolegend, San Diego, CA, USA). Approximately 70 µL of whole blood was stained for 20 min and then incubated for 15 min with FACS lysing solution (BD FACS Lysing; BD Biosciences, San Jose, CA) to lyse the erythrocytes. After two washes in an isotonic solution (Hemoton SPEC; Brazil), 500,000 events were acquired using a flow cytometer (LSR Fortessa; BD Biosciences, USA) and were analyzed using FlowJo Software (Tree Star, Ashland, OR, USA).

### Flow cytometry of *in vitro* TLR-activated cells

Peripheral blood mononuclear cells (PBMCs) were isolated by Ficoll-Hypaque density gradient (GE Health Care, Uppsala, Sweden) centrifugation. PBMCs (1.0 × 10^6^ cells/mL) were incubated with 10.0 μg/mL TLR3 agonist (Poly I:C hmw; InvivoGen, San Diego-CA, USA) or 5.0 μg/mL TLR7/8 agonist (CL097; InvivoGen), and CD107a PE-Cy5 (Pe.Cy5/Clone: H4A3) was added to detect degranulating NK cells. The samples were plated and incubated at 37 °C with 5% CO_2_ for 6 h. After 2 h of incubation, Brefeldin (10.0 µg/mL; Sigma, St. Louis-MO, USA) was added to the cultures for another 4 h of incubation. After incubation, the cells were washed and incubated with human IgG for 15 min, followed by incubation with Live-Dead reagent (Invitrogen, Eugene, OR, USA) for 20 min at room temperature. Cells were then subjected to fixation with Cytofix/Cytoperm solution (BD Bioscience, San Diego, CA, USA) for 20 min and permeabilization with Perm/Wash solution for 20 min at 4 °C. The cells were then stained with CD3 (BV605/Clone: SK7), CD19 (Horizon V500/Clone: HIB19), CD16 (APC-Cy7/Clone: 3G8), CD56 (Alexa 700/Clone: B159), CD62L (FITC/Clone: Dreg 56), CD38 (PE/Clone: HIT2), IFN-γ (Horizon V450/Clone: B27) and TNF (PE-Cy7/Clone: MAB11) antibodies. Next, the samples were washed with Perm/Wash buffer (BD Bioscience, San Diego, CA, USA) and diluted in isotonic solution. Fluorescence Minus One (FMO) controls were performed for all antibody panels to confirm proper compensation and define positive signals. Boolean gate arrays were created using FlowJo software. A total of 300,000 events were acquired and analysed by flow cytometry (LSR Fortessa, BD Biosciences, USA). These analyses determined the expression frequency of each cytokine based on all possible combinations of the 3 different cytokines. Analysis of polychromatic flow cytometry data was performed with the SPICE Program (version 2.9, Vaccine Research Center, NIAID, USA).

### Statistical analysis

All cytokine measurements were background subtracted, taking into account the frequency of cells producing cytokines in the absence of antigenic stimulation. Kruskal–Wallis tests with Dunn’s post-test were used to compare variables between HIV-1-infected individuals, ESN subjects and healthy controls. P ≤ 0.05 was considered statistically significant.

## Results

### Cytokine secretion by CD56^+^CD62L^+^ NK cells in serodiscordant couples

NK cells are an important component of innate immunity, and CD62L is differentially expressed during various stages of human NK cell maturation, with high CD62L expression on CD56^bright^ NK cells and heterogeneous CD62L expression on CD56^dim^ NK cells^7^. Therefore, we evaluated cytokine secretion by CD62L^+^ NK cells following activation with TLR3 or TLR7/8 agonists in serodiscordant couples. The cohort of serodiscordant couples reported an average of 13 years in a single relationship, in which the majority of HIV-infected partners were successfully treated with ART, except for 4 individuals with 179; 50,930; 51; and 17,511 copies of RNA/mL (Supplementary Table 1). These serodiscordant couples had persistent sexual exposure to HIV-1, albeit with low viral loads.

**Figure 1:**
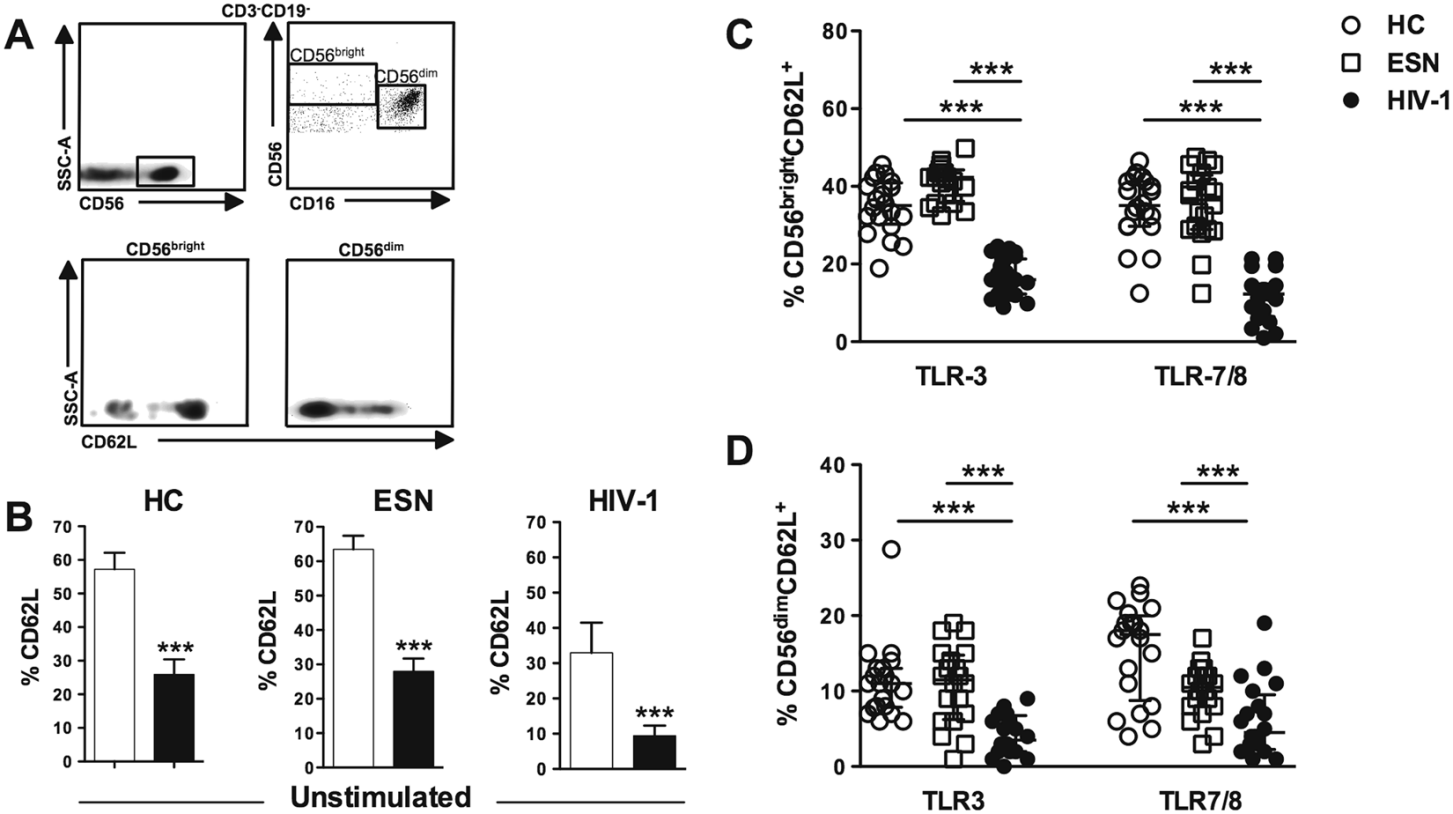
Decreased frequency of CD56^+^CD62L^+^ NK cells induced by agonists of TLR3 and TLR7/8 in HIV-1 individuals. (**A**) Gating strategy for CD62L expression in NK CD56^bright^ and CD56^dim^ cells from PBMCs. (**B**) Frequency of CD62L expression in NK CD56^bright^ (open bar) and CD56^dim^ (closed bar) cells in unstimulated PBMCs from healthy controls (HC, n = 20) and serodiscordant couples composed of ESN (n = 20) and HIV-1-infected individuals (n = 20). (**C**) CD56^bright^CD62L^+^ and (**D**) CD56^dim^CD62L^+^ cells assessed in PBMCs stimulated with an agonist of TLR3 (Poly I:C) or TLR7/TLR8 (CL097). Data represent the median and interquartiles. ***P ≤ 0.001.

Figure 1B shows that in unstimulated conditions, a higher frequency of CD62L expression was detected in CD56^bright^ than that in CD56^dim^ NK cells in all groups. After activation of PBMCs with TLR3 agonists (Poly I:C) or TLR7/8 agonists (CL097), we observed similar expression of CD62L in NK cell subtypes in the ESN and HC groups, whereas CD62L expression was markedly decreased in HIV-1-infected partners (Fig. 1C and D).

**Figure 2:**
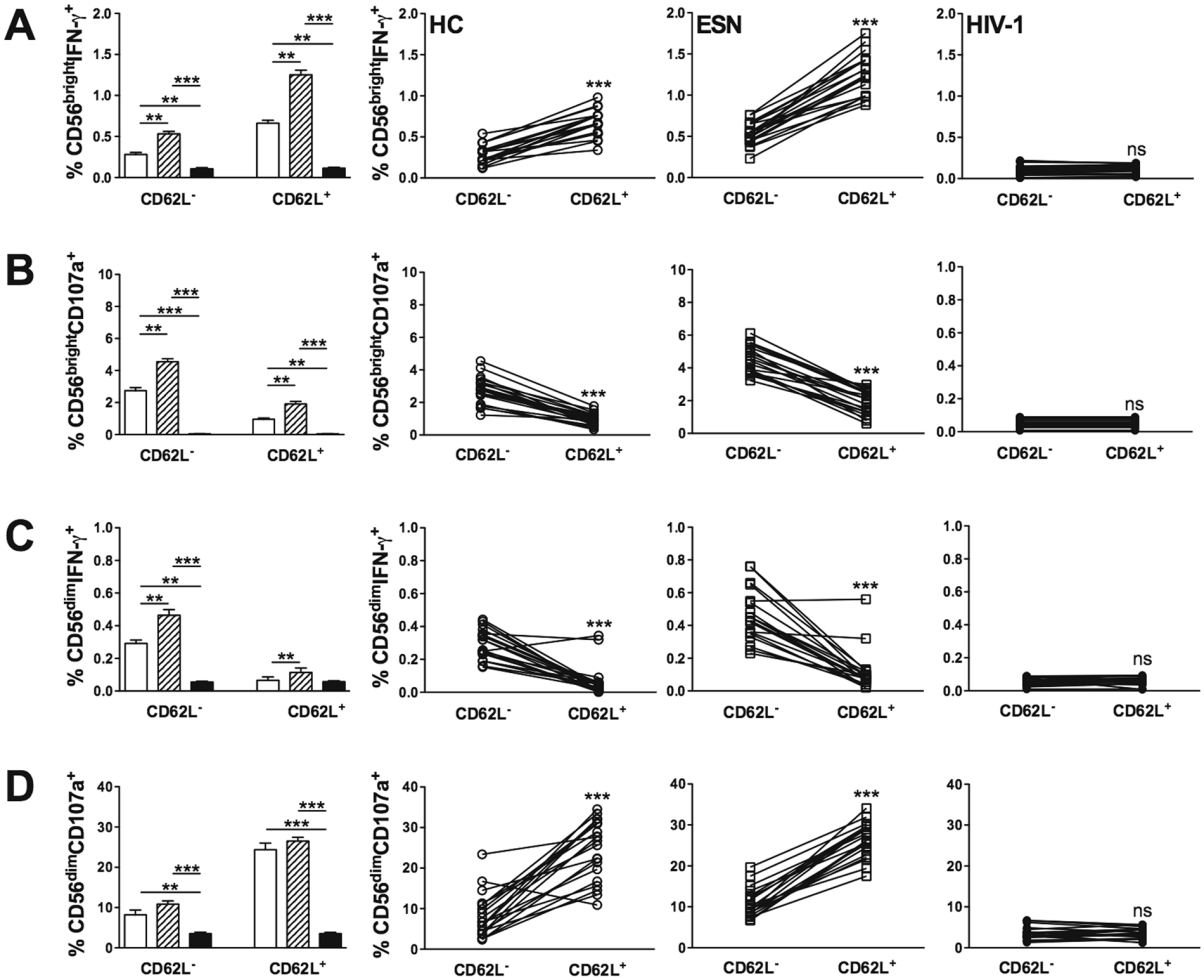
Increased IFN-γ and CD107a expression induced by TLR3 activation in CD62L^+^ NK cell subtypes of ESN individuals. Frequency of CD62L expression in (**A**) CD56^bright^IFN-γ^+^, (**B**) CD56^bright^CD107a^+^, (**C**) CD56^dim^IFN-γ^+^, and (**D**) CD56^dim^CD107a^+^ PBMCs stimulated with a TLR3 (Poly I:C) agonist from the HC (open bar, n = 20), ESN (crosshatched bar, n = 20) and HIV-1-infected (closed bar, n = 20) groups. Data represent the median and interquartiles. **P ≤ 0.01, ***P ≤ 0.001.

In evaluating cytokine secretion by NK cells, increased expression of IFN-γ and CD107a induced by TLR3 activation was detected in CD56^bright^ cells of the ESN group compared with that of the HC and HIV-1-infected groups, regardless of CD62L expression (Fig. 2A and B). In addition, IFN-γ secretion induced by the TLR3 agonist in CD56^dim^CD62L^−^ cells from ESN individuals was increased compared with that of the other groups (Fig. 2C). A similar frequency of CD56^dim^CD107a^+^ NK cells was observed in the ESN and HC groups (Fig. 2D). Of note, HIV-1-infected partners showed decreased CD107a expression and IFN-γ production in both NK cell subtypes (Fig. 2). CD62L expression was associated with the functional aspects of NK cell subsets, where CD56^bright^ cells secreted IFN-γ and CD56^dim^ cells expressed CD107a.

CD56^bright^CD62L^+^ NK cells activated by the TLR3 agonist showed increased IFN-γ production and decreased CD107a expression in both the ESN and HC groups, while the opposite effect was observed in CD56^dim^CD62L^+^ NK cells (Fig. 2). In contrast, the CD62L^+^ and CD62L^−^ NK cell response was barely detectable in the HIV-1-infected group (Fig. 2).

### ESN individuals exhibit higher polyfunctionality of CD56^bright^CD62L^+^ NK cells

Next, we evaluated the polyfunctional ability of CD56^bright^ NK cells to secrete TNF, IFN-γ and/or CD107a in response to TLR3 activation in the presence or absence of CD62L expression. The CD56^bright^ NK cell population was selected for analysis due to its higher expression of CD62L than that of CD56^dim^ cells. The gating strategy for polyfunctional CD56^bright^ NK cells is shown in Supplementary Figure 1.

**Figure 3:**
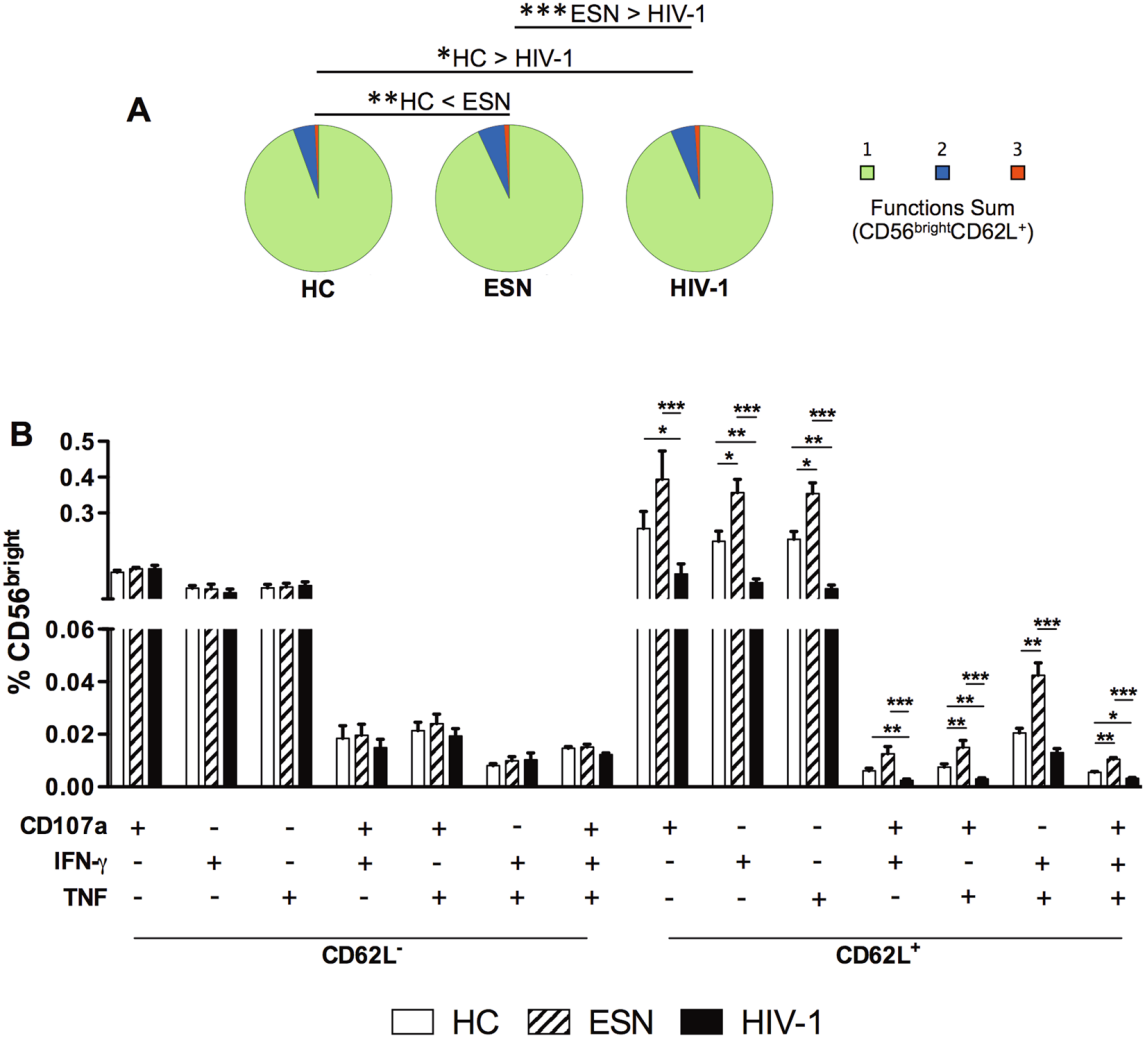
Polyfunctional responses of CD56^bright^CD62L^+^ NK cells induced by TLR3 activation in ESN individuals. (**A**) Pie charts represent the sum of 1, 2 or 3 cytokines secreted by CD56^bright^CD62L^+^ NK cells, following PBMC stimulation with TLR3 (Poly I:C) agonist; (**B**) Boolean analysis of CD56^bright^ NK cells secreting IFN-γ, CD107a and/or TNF according to CD62L expression in the HC (n = 20), EU (n = 20) and HIV-1-infected (n = 20) groups. *P ≤ 0.05, **P ≤ 0.01, ***P ≤ 0.001.

Figure 3 shows an increased frequency of CD56^bright^CD62L^+^ NK cells secreting CD107a, TNF and IFN-γ simultaneously in the ESN group compared with that of the HC and HIV-1-infected groups (Fig. 3A). These findings demonstrate that TLR3 activation enhanced the polyfunctional CD56^bright^ NK cell response in ESN individuals, principally with respect to CD62L^+^ cells, in contrast to the reduced NK cell function observed in HIV-1-infected partners (Fig. 3B).

**Figure 4:**
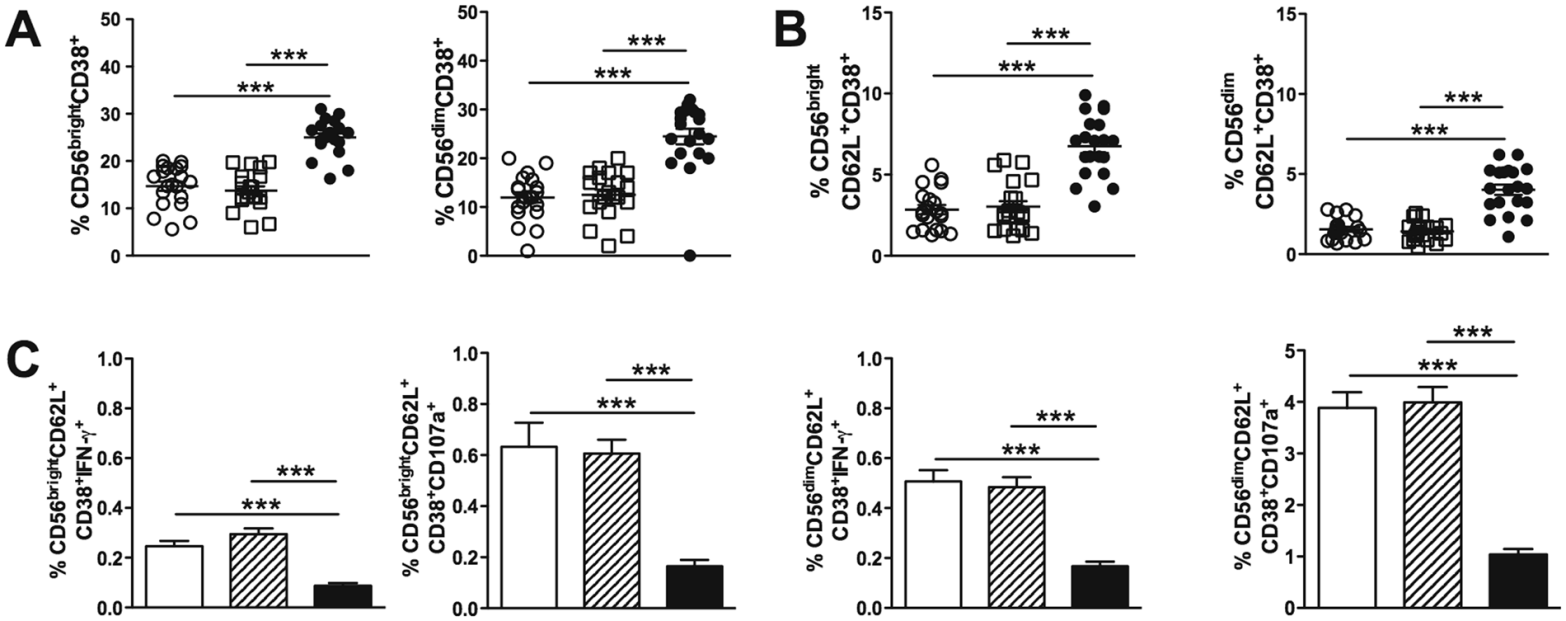
Impaired function of activated CD62L^+^CD38^+^ NK cells in HIV-1-infected individuals. (**A**) The frequencies of CD56^bright^ and CD56^dim^ CD38^+^ NK cells after PBMC stimulation with a TLR3 (Poly I:C) agonist were analysed for the HC (open circle, n = 20), ESN (open square, n = 20) and HIV-1-infected (closed circle, n = 20) groups. (**B**) Frequencies of CD56^bright^ and CD56^dim^ CD38^+^CD62L^+^ NK cells. (**C**) Frequencies of CD56^bright^CD38^+^CD62L^+^ and CD56^dim^CD62L^+^CD38^+^ cells secreting IFN-γ and expressing CD107a in the HC (open bar), ESN (crosshatched bar) and HIV-1 (closed bar) groups. ***P ≤ 0.001.

NK activation through CD38 of HIV-1-infected individuals is associated with viremia and disease progression markers^16, 17^, although CD38 expression in NK cells of ESN subjects has not been previously assessed. Higher CD38 expression after TLR3 stimulation was detected in CD56^bright^ and CD56^dim^ NK cells of the HIV-1-infected group compared with that of HC and ESN individuals (Fig. 4A), as well as in NK cells co-expressing CD38 and CD62L (Fig. 4B).

Although there was an increased frequency of CD62L^+^CD38^+^ NK cells in HIV-1-infected subjects, these cells showed a decreased functional ability compared with that of cells from HCs and ESN partners (Fig. 4C).

### Activated NK CD56^bright/dim^ cells expressing inhibitory/exhaustion markers in HIV-1 infected subjects

Since activated NK cells (CD38^+^) from HIV-1 individuals showed decreased IFN-γ/CD107a production induced by the TLR3 agonist, we next evaluated the expression of exhaustion/inhibition molecules, such as NKG2A, PD-1, and Tim-3, on NK cells.

**Figure 5:**
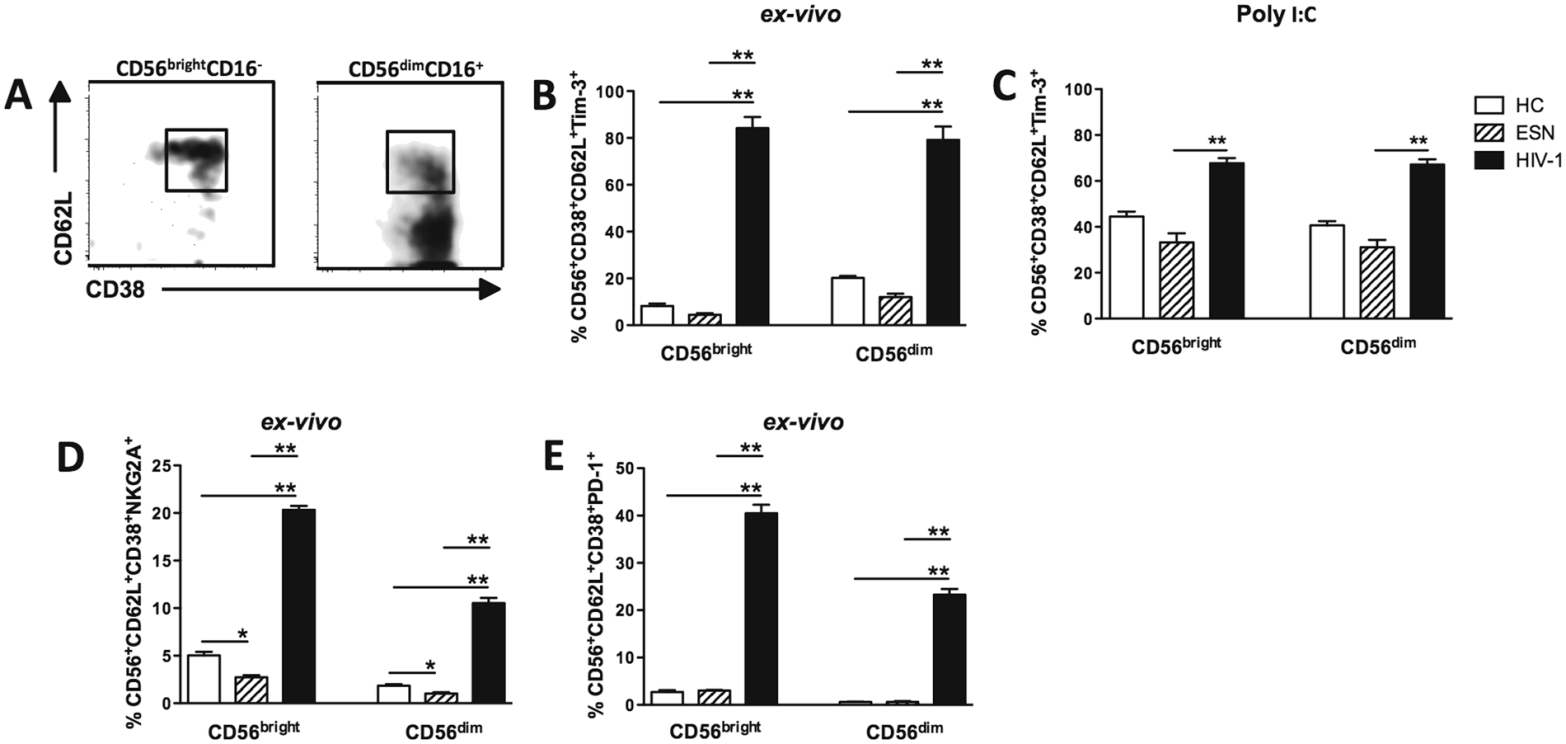
Expression of inhibition and exhaustion receptors in CD56^bright/dim^ CD62L^+^CD38^+^ NK cells in HIV-1-infected individuals. (**A**) Gating strategy in CD56^bright/dim^CD16^+^CD62L^+^CD38^+^ NK cells to determine inhibition/exhaustion receptor expression. (**B** and **C**) *Ex vivo* frequency of Tim-3 expression before and after TLR3 stimulation (Poly I:C) of PBMCs for 6 hours and (**D**) frequency of NKG2A and (**E**) PD-1 expression in the CD56^bright^ e CD56^dim^ NK cells of the HC (n = 5, open bar), EU (n = 5, crosshatched bar) and HIV-1-infected (n = 5, closed bar) groups. *P ≤ 0.05, **P ≤ 0.01.

Figure 5B–E shows high frequencies of Tim-3, NKG2A and PD-1 in NK CD56^bright/dim^CD62L^+^CD38^+^ cells in peripheral blood of HIV-1-infected individuals compared to those of the HC and ESN groups. Similar results were obtained by measuring the median fluorescence intensity (MFI) of these molecules (Supplementary Figure 2). Again, an increased frequency of CD56^bright/dim^CD62L^+^CD38^+^ NK cells expressing Tim-3 was induced by TLR3 stimulation only in the HIV-1 infected partners (Fig. 5C).

Although there was a small percentage of CD56^+^CD38^+^ NK cells expressing NKG2A in the uninfected groups, it was decreased in ESN individuals compared to those of the HC controls (Fig. 5D and Supplementary Figure 2).

These findings indicated that activated CD38^+^ NK cells express inhibitory/exhaustion molecules in HIV-1-infected partners, which may, in part, be responsible for the impaired TLR3 response.

## Discussion

In this study, we found that NK cells from ESN individuals were more responsive to TLR3 signalling with respect to the production of IFN-γ and expression of CD107a than those from HIV-1-infected partners or healthy subjects. In response to TLR3 or TLR7/8 agonists, the percentage of CD62L^+^ NK cells was similar between the ESN and HC groups, but was markedly reduced in the HIV-1-infected group. Moreover, an increased polyfunctional response induced by TLR3 activation was detected in CD56^bright^CD62L^+^ NK cells from ESN individuals. Along with the decreased percentage of NK cells in HIV-1-infected subjects and impaired IFN-γ and CD107a expression induced by TLR3 stimulation in CD38^+^ NK cells, we found high expression of inhibitory/exhaustion receptors, as Tim-3, PD-1 and NKG2A. In contrast, low activation and a strong response to TLR activation was detected in NK cells of ESN partners. The natural resistance to HIV-1 was mediated, in part, by NK cells with high TLR3 responsiveness, polyfunctionality and low inhibitory marker expression.

In the ESN and HC groups, CD62L expression in CD56^bright^ NK cells was associated with increased IFN-γ secretion, and CD107a production was increased in CD56^dim^ NK cells that also expressed CD62L. This profile was not observed in HIV-1-infected partners, in whom CD62L expression was markedly reduced even upon TLR3 or TLR7/8 activation. All the HIV-1-infected partners were receiving ART, and the majority (16 subjects) had an undetectable viral load, which was not sufficient to increase CD62L movement to the cell membrane. Interestingly, NK cells expressing receptors and co-receptors for HIV-1, including CD4, CCR5 and/or CXCR4, were persistently infected with HIV-1 in ART-treated subjects^30^. Moreover, HIV-1 accessory proteins, including Nef and Vpu, retain CD62L in perinuclear compartments to prevent its export to the plasma membrane^30, 31^.

The high TLR3 responsiveness of NK cells in ESN subjects was notable even with respect to the HC group. TLR3 induces the production of cytokines and cytotoxic molecules through a mechanism dependent on p38 MAPK activation in NK cells^32^. Although we did not study the signalling pathways downstream of TLR3, our data suggest that p38 MAPK may be activated more strongly in the ESN group. NK cells from ESN individuals produce IFN-γ and lyse K562 cells in response to activation with PMA and ionomycin^11, 12^. Another study showed that a TLR3 polymorphism, in which the leucine residue at position 421 is substituted for phenylalanine and confers receptor activation, was predominant in a cohort of drug users in Spain^33^. These ESN drug users also showed an increased response to TLR3 stimulation (Poly I:C), indicating that a common TLR3 allele confers immunologically mediated protection from HIV-1 and suggesting the potential use of TLR3 triggering in HIV-1 immunotherapy^33^.

CD56^bright^ NK cells are high producers of cytokines and constitute the subtype that predominantly expresses CD62L. Therefore, we evaluated the polyfunctional CD56^bright^ NK cell response induced by a TLR3 agonist in the presence and absence of CD62L expression. We verified that ESN individuals possess CD56^bright^ NK cells with an increased ability to secrete multiple cytokines, mainly cells that are CD62L^+^. The high polyfunctionality of NK cells expressing CD62L in ESN individuals could be related to resistance to HIV-1 infection. Characteristics of HIV-specific T cell responses, such as specificity, breadth, magnitude, polyfunctionality, and phenotype, may underlie the control of viral replication in HIV-1 infected individuals^34^. In addition, a high frequency of polyfunctional CD8^+^ NK cells is associated with slower disease progression in chronic HIV-1 infection^35^.

We previously demonstrated that PBMC activation with PMA and ionomycin increased the co-expression of IFN-γ and CD107a in CD56^bright^ and CD56^dim^ NK cells in ESN individuals, whereas expression was not affected in the HIV-1 group compared with expression in the HCs^28^. These data support the hypothesis that impairment of NK signalling in HIV-1-infected partners is related to TLR activation instead of the PKC pathway.

Usually, the CD38 ectoenzyme in T cells is an indicator of chronic cell activation during HIV-1 infection^36, 37^. CD38 and CD16 are functionally dependent and physically associated, and therefore, CD38 ligation promotes target cell killing by human NK cells^38^. Additionally, disease markers, including the CD4^+^ T cell percentage, viral load, and CD4^+^ T cell activation, have been associated with CD38 expression levels in both iNKT and NK cells^17^. Elevated levels of iNKT cell and NK cell activation are associated with viremia and disease progression markers in both HIV-1 and HIV-2 infections^17^. Moreover, in HIV-1-infected patients who achieve viral suppression following ART treatment, NK cell activation persists^39^. We showed that CD38 expression in NK cell subsets in the HIV-1-infected group was associated with an impaired ability to produce IFN-γ and CD107a. The co-expression of CD62L and CD38 in NK cells was also pronounced in HIV-1-infected individuals, suggesting that even immature NK cells can display an activated profile during infection. Whether the CD38^+^ NK cells are associated with immune activation as are CD4^+^ and CD8^+^CD38^+^ T cells in the chronically HIV-1-infected subjects^40, 41^ has yet to be determined. Curiously, low percentages of CD38^+^ NK cells were found in HC as well as ESN subjects, which showed a low activation profile.

The CD38 expression in NK cells subsets was associated with Tim-3, NKG2A and PD-1 expression and impaired functional ability in the chronically infected HIV-1 partners. NKG2A expression in the activated NK cells was decreased in the uninfected groups, predominantly in the ESN individuals; it was evaluated in NK cells expressing CD38, a population that is poorly represented in ESN subjects. In contrast, NK cells of a Vietnamese population of highly exposed uninfected drug users had a similar distribution of NKG2A and NKG2C expression among CD107a^+^ NK cells compared to that of HIV-1-infected individuals^42^. The difference in NKG2A expression in NK cells could be due to the intensity of HIV-1 exposure or route among the ESN cohorts. Our cohort is considered chronic, with low HIV-1 exposure, since the majority of HIV-1 infected partners were receiving ART treatment. Moreover, the decreased NKG2A expression in a small percentage of NK cells of ESN individuals may be correlated with the high TLR3 responsiveness since NKG2A is associated with suppression of cytotoxicity in HIV-1 patients with advanced clinical status^18^. Other exhaustion molecules, such as the PD-1 and Tim-3, were barely detected in NK CD38^+^ cells from ESN subjects and healthy controls. ESN and HIV-controllers have lower expression of CD69, LAG-3, PD-1, and Tim-3 in NK cells and CD4^+^ T cells compared to that of HIV progressors^43^. Our results showed for the first time that the low activation profile was due to a low frequency of NK CD38^+^ cells in ESN individuals and inhibitory/exhaustion molecule expression.

Together, our results show that ESN individuals possess NK cells with pronounced responsiveness to TLR3 activation and the ability to develop a polyfunctional response, as determined by cytokine production and degranulation. Our data support the hypothesis that the immune activation in chronically HIV-infected individuals affects the NK cells, leading for exhausted/inhibited profile. Protective immunity against HIV-1 infection in ESN individuals is associated with a low activation NK profile. Understanding the mechanisms underlying the activation of NK cells with TLRs agonists in naturally controlled HIV-1 infection may contribute to developing strategies that harness NK cells to treat HIV infection.

## Electronic supplementary material

Supplementary Information
