# Supplementary material for: Polyfunctional natural killer cells with a low activation profile in response to Toll-like receptor 3 activation in HIV-1-exposed seronegative subjects
Source: Sci Rep. 2017 Apr 3;7:524. doi: 10.1038/s41598-017-00637-3 (PMC5428831; doi:10.1038/s41598-017-00637-3)
Supplement: Supplementary file 1 — Supplementary Information [file 41598_2017_637_MOESM1_ESM.doc]

**Title: Polyfunctional natural killer cells with a low activation profile in response to Toll-like receptor 3 activation in HIV-1-exposed seronegative subjects**

**Authors:** Josenilson F. Lima^1^, Luanda M. S. Oliveira^1^, Nátalli Z. Pereira^1^, Alberto J. S. Duarte^1^, Maria N. Sato^1,*^

**Supplementary Table 1.** Demographic characteristics of serodiscordant couples and healthy control individuals.

| **Groups** | **N** | **Gender** | **Years** | **CD4 (per mm^3^)** | **CD8 (per mm^3^)** | **Time Diagnostic Yars** | **Time Relationship Yars** | **Viral Load (Copies RNA/mL)** |
| --- | --- | --- | --- | --- | --- | --- | --- | --- |
| HC | 20 | 7M/13F | 42.81 ± 2.12 | 983 ± 87.59 | 605 ± 72.74 | - | - | - |
| ESN | 20 | 10M/10F | 43.81 ± 2.14 | 1.309 ± 76.09 | 799.5 ± 90.62 | - | - | - |
| HIV-1 | 20 | 10M/10F | 42.85 ± 1.58 | 622 ± 87.99 | 3.578 ± 417.10 | 9.5 ± 1.23 | 13 ± 1.56 | <50 (4/20)* |

Results are shown as median and interquartiles. HC: Health Control, ESN: Exposed Seronegative, HIV-1: Infected HIV-1+. F: Female, M: Male. *Value represents detectable VL in 4/20 (179; 50,930; 51; 17,511; copies/mL).

**Supplementary Figure 1.** Gating strategy to polyfunctional CD56^bright^ NK cells analysis.

**Supplementary Figure 2.** Expression of inhibitors or exhaustion receptors in CD56^bright/dim^CD62L^+^CD38^+^ NK cells.
